# Coumarin-Based Profluorescent and Fluorescent Substrates for Determining Xenobiotic-Metabolizing Enzyme Activities In Vitro

**DOI:** 10.3390/ijms21134708

**Published:** 2020-07-01

**Authors:** Hannu Raunio, Olli Pentikäinen, Risto O. Juvonen

**Affiliations:** 1 School of Pharmacy, Faculty of Health Sciences, University of Eastern Finland, 70600 Kuopio, Finland; risto.juvonen@uef.fi; 2 Institute of Biomedicine, Faculty of Medicine, University of Turku, 20520 Turku, Finland; olli.pentikainen@utu.fi

**Keywords:** cytochrome P450, CYP, glucuronidation, sulfonation, methylation, fluorescence, molecular modeling, enzyme

## Abstract

Activities of xenobiotic-metabolizing enzymes have been measured with various in vitro and in vivo methods, such as spectrophotometric, fluorometric, mass spectrometric, and radioactivity-based techniques. In fluorescence-based assays, the reaction produces a fluorescent product from a nonfluorescent substrate or vice versa. Fluorescence-based enzyme assays are usually highly sensitive and specific, allowing measurements on small specimens of tissues with low enzyme activities. Fluorescence assays are also amenable to miniaturization of the reaction mixtures and can thus be done in high throughput. 7-Hydroxycoumarin and its derivatives are widely used as fluorophores due to their desirable photophysical properties. They possess a large π-π conjugated system with electron-rich and charge transfer properties. This conjugated structure leads to applications of 7-hydroxycoumarins as fluorescent sensors for biological activities. We describe in this review historical highlights and current use of coumarins and their derivatives in evaluating activities of the major types of xenobiotic-metabolizing enzyme systems. Traditionally, coumarin substrates have been used to measure oxidative activities of cytochrome P450 (CYP) enzymes. For this purpose, profluorescent coumarins are very sensitive, but generally lack selectivity for individual CYP forms. With the aid of molecular modeling, we have recently described several new coumarin-based substrates for measuring activities of CYP and conjugating enzymes with improved selectivity.

## 1. Background

### 1.1. Xenobiotic Metabolism

The metabolism of xenobiotics (foreign substances) in a living organism is vitally important to maintain homeostasis. Xenobiotics are transformed in enzyme-catalyzed oxidation, reduction, hydrolysis, and conjugation reactions to more hydrophilic and excretable metabolites. Usually these reactions yield metabolites with diminished biological activity, but sometimes reactive and more toxic intermediates are formed. In conjugation reactions, a cofactor (small endogenous molecule) is transferred to a functional group of a xenobiotic. The functional group is either already present or is created by reactions of oxidation, reduction or hydrolysis. Conjugations are highly important in overall metabolism because they usually inactivate xenobiotics or make reactive metabolites less harmful. In addition, conjugates are often substrates to transporters mediating excretion in the kidney and liver [1,2].

Cytochrome P450 (CYP) monooxygenases are the most versatile xenobiotic-metabolizing enzymes (XMEs), which oxidize all kinds of lipophilic agents entering the body. CYP-mediated metabolism is an essential mechanism of elimination for both xenobiotics and lipophilic endogenous compounds (endobiotics) such as steroid hormones, bile acids, and lipid-soluble vitamins. CYP enzymes are supported by NADPH CYP reductase, which provides electrons to activate oxygen in the catalytic oxidation reactions of xenobiotics. In all nature, CYP enzymes comprise a very large superfamily, with 18 families in mammals and 57 individual forms in the human genome. Of these CYP forms, ~10 members in families CYP1, CYP2 and CYP3 catalyze the oxidation of xenobiotics to a significant degree. CYPs are most abundantly expressed in the liver, but they are also present in other barrier tissues such as the gastrointestinal tract, lung, skin, nose epithelium, and kidney [3,4,5,6].

It is estimated that >90% of enzymatic reactions of all organic compounds (general chemicals, natural and physiological compounds, and drugs) are catalyzed by CYP enzymes. About 75% of CYP reactions of clinically used drugs can be accounted for by a set of five hepatic CYP forms: CYP1A2, CYP2C9, CYP2C19, CYP2D6, and CYP3A4 [7]. Conjugation reactions, especially glucuronidation by uridine diphosphate glucuronosyltransferases (UGTs) and sulfonation by sulfotransferases (SULTs), also play a major role in the elimination of xenobiotics and harmful endobiotics [6]. Rates of metabolic enzymes are modulated by numerous endogenous and exogenous factors, including genetic polymorphisms, sex, age, ethnicity, general health conditions, and induction and inhibition by xenobiotics [4,8].

When new chemical entities, especially drugs, biocides, and food additives, are developed, it is essential to evaluate their metabolic pathways in target species and the major XMEs involved. Especially for drugs it is necessary to establish the kinetics and possible inhibition and induction of the XMEs involved.

### 1.2. Methods to Measure XME Activities In Vitro

After performing an in vitro enzyme reaction, formation of metabolites or decrease of substrate can be detected by absorption of light (spectrophotometry), emitted fluorescence (fluorometry), mass/charge ratio (mass spectrometry, MS) or light pulses produced by a radioactive label. For the measurement of a substrate or its metabolites, the most versatile analytical approaches today are liquid chromatography (LC)-MS methods. The drawback of these methods is that the equipment is expensive, their use is labor intensive, and capacity may be saturated [9,10].

Fluorescence is a phenomenon where a molecule (fluorophore) absorbs and then emits lower-energy light. Fluorophores usually contain several conjugated aromatic groups or cyclic molecules with several π bonds or nonbonding electrons. The key parameters in this process are the absorption maxima (λ_max_) (excitation) and the extinction coefficient at λ_max_ (ε, emission). Loss of energy occurs in excitation due to rapid relaxation to the first singlet excited state (S_1_) and reorganization of solvent molecules around the altered dipole of the excited state. Fluorescence arises when this excited state is relaxed to the ground state (S_0_) by emission of a photon. Each fluorophore has a characteristic pair of excitation (λ_max_) and emission maximum (λ_em_). Chemiluminescence is similar to fluorescence but differs from it in that the electronic excited state is the product of a chemical reaction rather than of the absorption of a photon [11,12].

Fluorescence methods are widely used to evaluate rates and kinetic mechanisms of enzyme reactions. Fluorophore-based enzyme assays are usually highly sensitive and specific. This is particularly valuable when performed on small specimens of tissues or other sources with low enzyme activities. Fluorescence assays are also amenable to miniaturization of the reaction setup and high throughput. Thus, further increases in sensitivity can be achieved with a decrease in demand for enzyme source and fluorescent substrate [13,14,15]. An advantage of fluorophore-based enzyme assays is also that both fixed time and online continuous assays (milliseconds to hours) can be applied for multiple purposes. 

**Figure 1:**
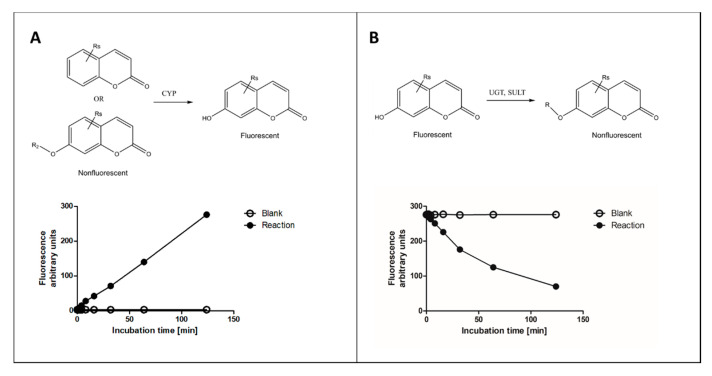
Principles of fluorescence-based cytochrome P450 (CYP) oxidation and conjugation assays with coumarin derivatives. In CYP oxidation, a nonfluorescent coumarin is oxidized to corresponding fluorescent 7-hydroxycoumarin (panel **A**), which can be conjugated to a nonfluorescent glucuronide or sulfate by uridine diphosphate glucuronosyltransferase (UGT) or sulfotransferase (SULT) enzymes, respectively (panel **B**). The activities of enzymes can be determined from fixed time (end-point) or continuous assay (kinetic) setups.

Of the various fluorophore-based approaches, we focus here on those that measure fluorescence change of coumarin substrates in reactions catalyzed by several types of XMEs, with main emphasis on our recent experience. In these reactions, a nonfluorescent coumarin derivative is metabolized to corresponding fluorescent 7-hydroxycoumarin metabolite(s) or vice versa (Figure 1). Other fluorophores commonly used for determining activities of XMEs include alkoxyresorufins [16], quinolines [17], furanones [14,17] and some proprietary ones such as Vivid® fluorogenic substrates [18].

### 1.3. Coumarins as Substrates for XMEs

**Figure 2:**
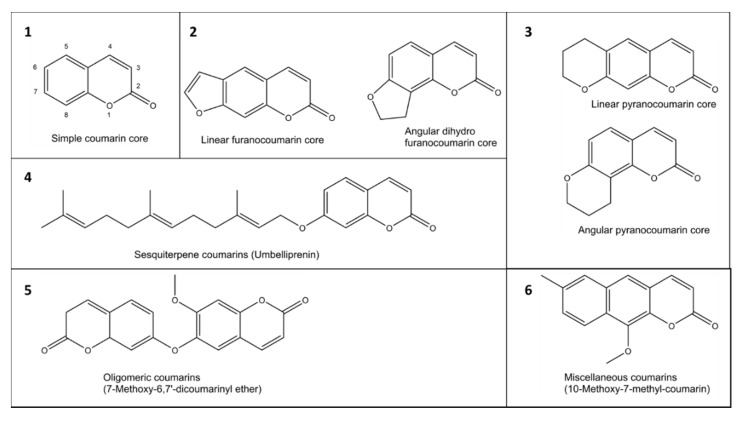
Examples of six groups (**1**–**6**) of coumarins in plants.

The coumarin (2H-1-benzopyran-2-one) scaffold is made of fused benzene and α-pyrone rings. Coumarins can be grouped in six types based on their extended backbone structures (Figure 2): (1) simple coumarins, (2) furanocoumarins, (3) pyranocoumarins, (4) sesquiterpenyl coumarins, (5) oligomeric coumarins and (6) miscellaneous coumarins [19]. The coumarin scaffold occurs in constituents of food and numerous natural and synthetic products such as drugs, perfumes, rodenticides, and laser dyes [19,20].

Coumarin is widely used as a fluorophore due to its desirable photophysical properties. Coumarin possesses a large π-π conjugated system with electron-rich and charge transfer properties. This conjugated structure leads to their applications as fluorescent sensors for biological activities. Substitution at the 7-position with electron-donating groups yields highly fluorescent molecules, 7-hydroxycoumarin (umbelliferone) being the core structure of these molecules. Many suitable combinations of substitutions at the 7-hydroxycoumarin scaffold do not destroy the intense fluorescence [11,21]. However, we have noticed that 4, 5-dialkyl substituents suppress the fluorescence intensity to a minimum (unpublished data), and fluorescence of 7-hydroxywarfarin is low. Coumarin and many of its derivatives are converted to 7-hydroxycoumarin metabolites in the typical oxidation reaction of CYP enzymes (Figure 1).

The fluorescent properties of 7-hydroxycoumarin were already used in the 1950s to evaluate its metabolism in the laboratory of R.T. Williams. It was observed that, although fluorescence of 7-hydroxycoumarin was strong in ultraviolet light at pH ~10, its glucuronide and sulfate conjugates showed little or no fluorescence. Based on this, a sensitive fluorescence method was developed for the determination of β-glucuronidase activity, in which nonfluorescent glucuronide conjugate was hydrolyzed to fluorescent 7-hydroxycoumarin [22]. An in vitro method for measuring coumarin 7-hydroxylation activity using fluorescence detection was subsequently developed for CYP enzymes in the same laboratory. This study also confirmed the marked interspecies differences in hepatic coumarin 7-hydroxylation capacity [23]. A simplified version of the 7-ethoxycoumarin O-deethylation to 7-hydroxycoumarin assay was published by Aitio and colleagues in the late 1970s [24]. This method became popular and is still being used in fixed time (end-point) enzyme assays.

**Figure 3:**
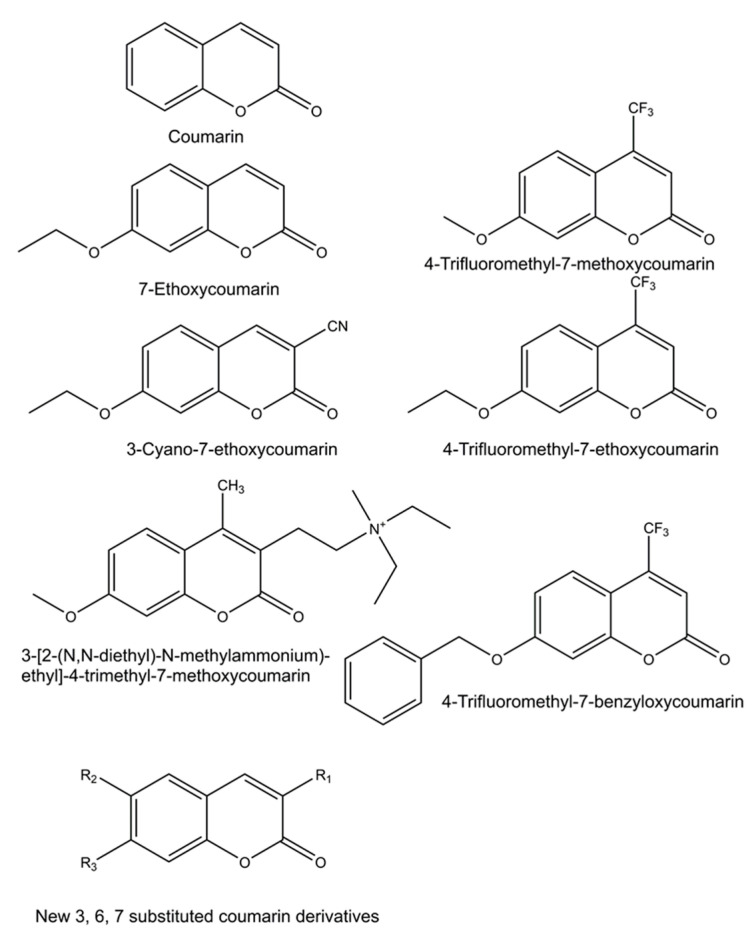
Structures of profluorescent CYP substrates oxidized to fluorescent 7-hydroxycoumarin derivatives. New coumarin derivatives can have various combinations of substituents at position 3 (3-hydroxyphenyl, 3-methoxyphenyl, 3-benzyloxo, 4-fluorophenyl, 4-trifluoromethylphenyl or pyridyl); at position 6 (hydrogen, methyl, methoxy, chloro or hydroxy); and at position 7 (hydrogen or methoxy).

Numerous new 7-substituted coumarin derivatives for measuring activities of CYP enzymes have been introduced since the 1970s. These include 7-ethoxycoumarin, 3-cyano-7-ethoxycoumarin (CEC), 7-ethoxy-4-trifluoromethylcoumarin (EFC), 7-methoxy-4-trifluoromethylcoumarin (MFC), 7-methoxy-4-aminomethylcoumarin (MAMC), 3-[2-(N,N-diethyl-N-methylammonium)ethyl]- 7-methoxy-4-methylcoumarin (AMMC), and 7-benzyloxy-4-trifluoromethylcoumarin (BFC) (Figure 3). These alkylated derivatives of 7-hydroxycoumarin are ‘blocked’ substrates, whose fluorescence signal is minimal compared to the 7-O-dealkylated oxidation product. Some of these have been commercialized for use in CYP-mediated drug interaction studies, pioneered by the Gentest Corporation (now Corning) [13,17,25]. A major shortcoming of these coumarin derivatives is that, with few exceptions, they are not selective but oxidized by several human CYP forms [13,17,25,26,27]. Coumarin itself is an example of a highly selective CYP substrate, as it is oxidized practically exclusively by human CYP2A6 to fluorescent 7-hydroxycoumarin [28,29]. In addition to being excellent substrates, numerous coumarins are potent CYP inhibitors, particularly furanocoumarins such as methoxsalen [30,31,32].

Ideally, sensitive and selective fluorophore substrates for different forms of XMEs would provide effective and low-cost tools for studying form-specific metabolism in simple assays using all kinds of enzyme sources, including whole tissue or subcellular fractions. However, although many fluorophore substrates are available, new substrates are needed as most human XMEs are still missing the optimal substrate, especially regarding selectivity for different enzyme forms. The flexible chemistry of the coumarin core offers diverse options for functionalization to design novel fluorescent substrates.

As part of an effort to find novel coumarin-based, pharmacologically active molecules, we have synthesized multiple derivatives of the coumarin scaffold. The synthesis protocols were kept simple and robust, and inexpensive starting materials were used. The detailed protocols are published [33,34,35]. In a typical procedure, a mixture of a salicylaldehyde derivative, a phenylacetic acid derivative, acetic acid anhydride and trimethylamine is heated at 100°C. After cooling, NaHCO_3_ solution is added, and the precipitate is filtered, dried, and recrystallized. The acetyl group(s) are removed by treating the compound with methanol/NaOH solution. The solution is acidified with HCl, and the precipitate is filtered and recrystallized if needed. Based on the elemental analysis and/or ^1^H-NMR the purity of compounds is typically >95%.

### 1.4. Establishing Fluorescence Assays—Practical Aspects

When setting up fluorescence assays to quantify enzyme activities, the first step is to establish incubation conditions supporting the reaction. Phosphate or Tris-HCl buffers are commonly used to gain the optimal ion concentration and pH of 7.4. All enzymes require their cofactors: NADPH for CYP oxidations, uridine 5’-diphosphoglucuronic acid (UDPGA) for UGTs, 3’-phosphoadenylyl sulfate (PAPS) for SULTs and S-adenosylmethionine (SAM) for COMT. The fluorescent substrates must have sufficient aqueous solubility (at least µM range), efficient metabolite formation, low background fluorescence, high signal-to-noise ratio, and appropriate excitation and emission wavelengths in the UV range [12,36].

During incubation a metabolite is formed, and the amount of substrate is decreased. Fluorescent 7-hydroxycoumarins are excellent for measuring substrate decrease, because several conjugating enzymes transfer a substituent to the 7-hydroxyl group making the product nonfluorescent (Figure 1B). A practical advantage of measuring conjugating reactions is that 7-hydroxycoumarins act as standards, allowing for reliable and accurate quantification of the reaction, whereas CYP oxidation reactions require the availability of a selective reaction standard for exact quantitation. However, in the latter case surrogate standards such as 7-hydroxycoumarin can be applied.

The solubility of coumarin in water is very high (20 mM). Lipophilic substituents decrease solubility, causing much variability to solubility among coumarin derivatives. The solubility of 7-hydroxycoumarins is higher than that of the corresponding derivatives. In our experience, even the poorest soluble coumarin derivatives have been soluble at µM concentrations. We routinely use 0.05–10 µM concentrations in the assays. Enzyme sources (especially tissues) and cofactors can produce background fluorescence. Contamination of tissue samples with blood causes problems because hemoglobin absorbs light efficiently at 370–450 nm. Too high concentrations of enzyme sample reduce fluorescence due to absorption of excitation or emission light and produce insufficient signal-to-noise ratio in the reaction. In the continuous (kinetic) assays up to 5% tissue in the reaction mixture does not excessively suppress fluorescence. Recombinant or purified enzymes added in small volumes in the incubation mixture yield no increase in the background. Tissue samples can be removed in the fixed time measurements by precipitation, but this adds extra steps. When very low activities are measured in fixed time assays, small differences in composition between sample and negative control may cause background fluorescence even after precipitation. In continuous assays background fluorescence is usually not a major issue, as fluorescence change is monitored against background during the entire measurement period.

Although both the excitation and emission peaks are broad for fluorescent compounds, it is important that these are optimized in the assays. Excitation wavelengths typically range from 300 to 420 nm and emission wavelengths from 350 to 500 nm for 7-hydroxycoumarin derivatives. Coumarins are also fluorescent; however, their excitation wavelengths are lower than 7-hydroxycoumarins. NADPH is strongly fluorescent <390 nm if excitation ~350 nm is used, but it is nonfluorescent at excitation >400 nm. The cofactors UDPGA and PAPS do not cause background fluorescence. Using excitation >400 nm minimizes background fluorescence and gives high enough emission fluorescence at 450–500 nm for coumarin-based assays. Negative control incubations lacking the enzyme, substrate or cofactor are needed to monitor background fluorescence.

When selecting the enzyme source, one must keep in mind that tissues and their cellular subfractions contain several forms of XMEs, most notably the liver. Since almost all fluorescent probes are not selective or lose selectivity at high substrate concentrations, heterologously expressed individual XMEs should be used when enzyme-specific reactions are studied. Nowadays, there is a good commercial supply of expressed human CYP, UGT and SULT enzymes.

To understand an enzyme reaction and its physiological role, it is essential to determine the effect of substrate concentration on the reaction rate (K_m_—the substrate concentration giving half-maximum rate) and the limiting rate for the reaction (V_max_—the rate at saturating substrate concentration). Experiments can then be rationally planned for evaluating potential inhibitors and characteristics of inhibition, including inhibitor potency and whether the inhibition mechanism is reversible, irreversible, competitive, un-competitive (catalytic) or mixed type of inhibition [12,36]. When interpreting the results, it should be considered that especially CYP3 enzymes may deviate from Michaelis–Menten kinetics with several probes, including fluorescent substrates [37,38]. In summary, sensitivity of fluorescence-based assays consists of the fluorescence properties of the substrates, products, cofactors, amount and purity of samples, and incubation time. All can be optimized as described here.

## 2. Use of Coumarin Substrates in Studying XMEs

### 2.1. CYP Enzymes

#### 2.1.1. CYP1 Family

The human CYP1 family comprises three enzymes: CYP1A1, CYP1A2, and CYP1B1, which differ particularly in expression patterns, CYP1A1 and CYP1B1 being extrahepatic enzymes. The amino acid sequence of CYP1A2 is 72% identical to that of CYP1A1, while CYP1B1 has lower amino acid sequence identity with both CYP1A1 (38%) and CYP1A2 (37%). All CYP1 family enzymes have a lot of similarity in shape and size of the active sites, leading to marked overlap in substrate preference (such as polycyclic aromatic hydrocarbons, heterocyclic aromatic amines, and estradiol) [39,40]. CYP1A1, CYP1A2, and CYP1B1 show 7-alkoxycoumarin dealkylation activities but do not show coumarin 7-hydroxylation activity. These human enzymes catalyze coumarin epoxidation to the toxic 2,3-epoxide at much lower rates than the corresponding rodent enzymes. EFC deethylation is catalyzed by these three enzymes in the order of CYP1A1 > CYP1B1 > CYP1A2 [13,31]. 7-ethoxycoumarin O-deethylation is catalyzed by CYP1A1 >> CYP1A2 ≈ CYP1B1 (unpublished results).

We recently developed 10 novel profluorescent coumarin derivatives with various substitutions at carbons 3, 6, and 7 [41]. All derivatives were oxidized to corresponding fluorescent 7-hydroxycoumarin metabolites. Accurate determination of the key enzyme kinetic parameters by individual human CYP forms was feasible with these substrates. CYP1 family enzymes converted four of the coumarin derivatives to fluorescent metabolites, CYP1A2 being a selective catalyst of 6-methoxy-3-(4-trifluoromethylphenyl) coumarin. In silico analysis revealed that simple 3-phenylcoumarins target CYP1 forms and CYP2A6. Hydrogen bonding from the coumarin carbonyl to Ser122 or Thr124 residues in CYP1A1 or CYP1A2, respectively, orientates position 7 of the coumarin core towards the heme, while the 3-phenyl interacts with the CYP1 conserved Phe224.

We used some of these novel CYP1 substrates, along with the prototype substrate 7-ethoxyresorufin, to characterize inhibition of human CYP1A1, CYP1A2 and CYP1B1 enzymes by α-naphthoflavone (ANF) and the novel inhibitor N-(3,5-dichlorophenyl) cyclopropanecarboxamide (DCPCC) [42].

#### 2.1.2. CYP2 Family

There are five major liver CYP2 subfamilies with individual XMEs (CYP2A, CYP2B, CYP2C, CYP2D, and CYP2E) [8].

**CYP2A subfamily.** The highly similar hepatic CYP2A6 and extrahepatic CYPA13 forms differ in only 32 amino acids, of which ten are in their relatively small active sites. Typical substrates are low-molecular-weight compounds containing 2 hydrogen bond acceptors [39,43,44]. Both enzymes catalyze coumarin 7-hydroxylation and 7-alkoxycoumarin dealkylation of coumarins such as 7-methoxycoumarin and 7-ethoxycoumarin. The oxidation of 7-ethoxycoumarin and 7-methoxycoumarin produces both 7-hydroxycoumarin and 3-hydroxycoumarin by CYP2A6 and CYP2A13. CYP2A6 and CYP2A13 do not produce 3-hydroxycoumarin in coumarin oxidation [31]. CYP2A6 is the only enzyme in the human liver catalyzing coumarin 7-hydroxylation to a significant degree, and consequently, the formation of 7-hydroxycoumarin can be used as an in vitro and in vivo probe for CYP2A6 activity [28,29]. It is well established that the metabolism of coumarins by CYP and conjugating enzymes is species-dependent. High doses of oral coumarin administered evoke liver toxicity in rodents, especially in rats and some strains of mice. Coumarin is rapidly metabolized to the nontoxic 7-hydroxycoumarin by CYP2A6 in humans, CYP2A19 in pigs and by CYP2A5 in mouse strains expressing high levels of this enzyme in the liver. In many other species, including the rat, coumarin is converted to the proximate hepatotoxic metabolite coumarin 3,4-epoxide catalyzed by other CYP enzymes. Thus, species-specific liver toxicity is directly related to the pathways of coumarin metabolism [29,45].

6-Methylcoumarin is a novel substrate for human CYP2A6 and mouse CYP2A5 and pig CYP2A19 enzymes [46]. 6-Methylcoumarin is oxidized to fluorescent 7-hydroxy-6-methylcoumarin. Docking and molecular dynamics simulations of 6-methylcoumarin and 7-methylcoumarin in the active sites of CYP2A6 and CYP2A5 demonstrated a favorable orientation of the 7-position of 6-methylcoumarin towards the heme moiety. Different orientations of 7-methylcoumarin were possible in CYP2A6 and CYP2A5, making it an irreversible inhibitor for CYP2A6 and a reversible one for CYP2A5.

CYP2A6 is the principal enzyme metabolizing nicotine to inactive products [47]. Genetic variation in CYP2A6 is known to alter many smoking behaviors. People with a reduced or total loss of CYP2A6 activity are more likely to be non-smokers than those with normal CYP2A6 activity. Slower nicotine metabolizers may benefit most from nicotine replacement therapy due to a safer side effect profile and lower cost, whereas faster metabolizers, those with greater CYP2A6 activity, may benefit from the more expensive prescription drugs bupropion or varenicline [48]. Decrease of CYP2A6 activity by phenocopying the reduced activity genotype with chemical inhibitors could help smokers to reduce smoking and also help them quit [49]. In search of such inhibitors, we have used the high-throughput coumarin 7-hydroxylation assay as a surrogate marker for nicotine oxidation [50]. The most potent novel inhibitors found were thienopyridine derivatives with hydrogen bond acceptor atoms either in the core structure or the substituent, or in both. These inhibitors are among the most potent identified to date [51].

Scoparone is a bioactive coumarin derivative found in multiple plants. It is the major active component in the Chinese herbal medicine Yin Chen Hao, a component in many traditional Chinese medicine (TCM) products [52]. We developed a fluorescent-based assay method to evaluate the rate of scoparone 7-O-demethylation to scopoletin in various species [53]. The rate was higher in liver microsomes from pigs, mice, and rabbits than in those of humans. The human extrahepatic enzymes CYP1A1 and CYP2A13 were efficient catalysts of scoparone 7-O-demethylation. Mouse, rabbit and pig liver contained CYP2A enzymes efficiently catalyzing the reaction. According to these results, mouse, rat, pig, and rabbit are not appropriate surrogate species for evaluating scoparone metabolism and thus its pharmacokinetics in humans. 

In a follow-up study [54] we demonstrated, using LC-MS and fluorescence-based assays, that the major metabolic pathway of scoparone to isoscopoletin is 6-O-demethylation in liver microsomes from humans, mice, rats, pigs, and dogs. In contrast, 7-O-demethylation to scopoletin was the main reaction in rabbit. Of individual human CYPs, CYP2A13 exhibited the highest rate of isoscopoletin and scopoletin oxidation, followed by CYP1A1 and CYP1A2. Glucuronidation of isoscopoletin and scopoletin was catalyzed by the human UGTs 1A1, 1A6, 1A7, 1A8, 1A9, 1A10, and 2B17. The major in vivo metabolites of scoparone in humans were isoscopoletin glucuronide and sulfate conjugates.

**Figure 4:**
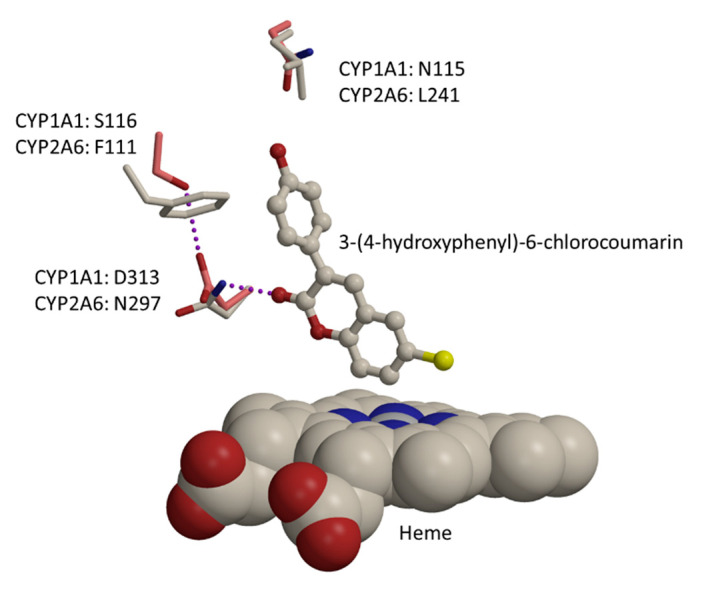
Size, shape, and electrostatic properties differ between CYP1 family enzymes and CYP2A6, affecting the binding preference of 3-phenyl coumarins, here a docked pose of 3-(4-hydroxyphenyl)-6-chlorocoumarin. In CYP2A6, Asn297 hydrogen bonds to the lactone group of coumarin derivative, while in CYP1 family enzymes (CYP1A1 shown here) there is a differently oriented aspartic acid at that sequence position. Furthermore, there are many other sequence differences that reflect into binding affinity and specificity, e.g., CYP2A6 has Leu241 vs CYP1A1 Asn115. Heme is shown as a cpk-model, bound substrate as ball-and-stick representation, amino acids as sticks (CYP1A1: pink carbon atom; CYP2A6: grey carbon atoms), and hydrogen bonds are shown as purple dotted lines.

CYP1 and CYP2A enzymes have overlapping substrate preferences. For example, four novel small 3-phenylcoumarins were metabolized efficiently (high intrinsic clearance) by both CYP2A6 and CYP1 enzymes [41]. The CYP1 family enzymes and CYP2A6 have an optimal size of the active site for catalysis of 3-phenylcoumarins. The catalytic sites of these CYPs are very hydrophobic, and accordingly, the binding of 3-phenylcoumarins is largely driven by the shape, but also polar interactions can contribute to binding. For example, in CYP2A6 there is an asparagine, Asn297, which donates a hydrogen bond to the lactone group of 3-phenylcoumarin (Figure 4). This particular orientation for Asn297 is forced by bulky Phe111. In this binding orientation, the C7 of coumarin core is placed next to the heme iron (Figure 4), and thus C7 is coordinated favorably for hydroxylation. In contrast, all human CYP1 family enzymes have an aspartic acid (CYP1A: Asp313; CYP1B1: Asp326) instead of asparagine at that sequence position (Figure 4). This aspartic acid is also oriented differently to Asn297 of CYP2A6, due to an intramolecular hydrogen bond with Ser116 (Figure 4). Accordingly, the lactone-group of 3-phenylcoumarins cannot be stabilized similarly to CYP1A family enzymes as in CYP2A6. In general, CYP2A6 favors smaller ligands than CYP1 family enzymes, as it has more bulky phenylalanine residues at the binding site (data not shown). Particularly for 3-phenylcoumarins, a significant difference in polarity of binding sites is seen close to the 4’-position of 3-phenyl, where there is an asparagine present in all CYP1 enzymes (Figure 4; CYP1A1: Asn115; CYP1A2: Asn117; CYP1B1: Asn126), while CYP2A6 has a similar-sized hydrophobic residue at that position (Figure 4; Leu241).

CYP2B subfamily. Only CYP2B6 is functional in humans in the CYP2B subfamily. EFC is an example of a coumarin metabolized efficiently by CYP2B6 [26]. Ticlopidine and clopidogrel are good inhibitors of CYP2B6 [32]. We used EFC as the substrate for evaluating novel inhibitors of CYP2B6. Out of a library of chemically diverse compounds screened, three pyridine derivatives were inhibitory at submicromolar concentrations: 4-benzylpyridine (BP), 4-(4-chlorobenzyl)pyridine (CBP), and 4-(4-nitrobenzyl)pyridine (NBP). Three-dimensional quantitative structure-activity relationship (3D-QSAR) analysis with the comparative molecular field analysis (CoMFA) method showed electrostatic and steric fields of CYP2B6 that are consistent with the structure of BP [55]. Subsequently, another group of investigators co-crystallized CYP2B6 with BP and CBP. Analysis of the structure showed that recognition of the benzylpyridines in the closed conformation of CYP2B6 is based solely on hydrophobicity, size, and shape [56].

CYP2C subfamily. The human CYP2C subfamily members are CYPs 2C8, 2C9, 2C18, and 2C19. Especially CYP2C9 metabolizes coumarins, including coumarin anticoagulant drugs such as S-warfarin, acenocoumarol, and phenprocoumon [8]. 7-hydroxywarfarin is weakly fluorescent. We found two novel coumarins, 3-(3-methoxyphenyl)coumarin and 3-(3-methoxyphenyl)-7-methoxycoumarin, which are oxidized more efficiently by CYP2C19 than the classical substrate CEC. However, these had some overlap with other CYPs, particularly with CYP1A1/1A2 and CYP2D6. Molecular docking showed that the 3’-O-methoxy oxygen in these compounds hydrogen-bond with Asn107 residue in CYP2C19 by replacing the water molecule present in the crystal structure [41].

CYP2D subfamily CYP2D6, the only active gene from this subfamily in humans, metabolizes 15-25% of clinically used drugs particularly nitrogen atom containing compounds [8]. CYP2D6 catalyzes dealkylation reactions on 7-alkoxycoumarins such as AMMC, MAMC and MFC, all of which are also catalyzed by several other CYP forms [26,31]. However, AMMC exhibits some degree of CYP selectivity, as CYP1A1, CYP1A2, CYP1B1, and CYP2B6 oxidize it only weakly [57]. In our series of novel coumarin substrates, 7-methoxy-3-(3-methoxyphenyl)coumarin was oxidized efficiently by CYP2D6. Molecular modeling showed that Ser304 at the enzyme active site could act as a hydrogen bond donor to orient this compound for 7-O-demethylation [41].

CYP2E subfamily human CYP2E1 metabolizes mainly small polar molecules such as ethanol but also has some larger aromatic substrates, including coumarins. Metabolism of coumarins by CYP2E1 leads to epoxidation at the 3,4 positions, dealkylation at the 7 position, and/or hydroxylation at various positions [31]. MFC is a nonselective substrate for CYP2E1 [26]. We developed a simple assay for measuring MFC O-demethylation activity, based on detection of fluorescence emitted by the metabolite 7-hydroxy-4-trifluoromethylcoumarin (HFC). This assay was used to determine the inhibition potency of structurally diverse compounds against human CYP2E1 [58].

CYP2J2 mediates formation of epoxyeicosatrienoic acids from arachidonic acid. In addition, it metabolizes a variety of structurally diverse drugs, including some antihistamines, anticancer agents, and immunosuppressants. However, no coumarin derivatives have yet been identified as CYP2J2 substrates [59].

#### 2.1.3. CYP3 Family

The main human CYP3 family enzymes are CYP3A4, CYP3A5, CYP3A7 and CYP3A43, of which hepatic CYP3A4 is the most important enzyme in the metabolism of xenobiotics, especially clinically used drugs. CYP3A4 and CYP3A5 have large active sites (~1400 Å3), >85% amino acid sequence similarity, and can metabolize large lipophilic molecules [8]. A good profluorescent CYP3A substrate has not yet been available. CYP3A4 is one of the several CYPs known to debenzylate BFC [26,60], but its oxidation rate is not high and relatively large amounts of CYP3A enzyme are needed to perform assays. We identified 3-((3-benzyloxo)phenyl)-7-methoxycoumarin as a new profluorescent substrate of CYP3A4 with higher catalytic rate than with BFC, but it is not selective as it is also oxidized by other CYP forms [41].

### 2.2. Conjugating Enzymes

#### 2.2.1. UGT Enzymes

The UGTs catalyze ~35% of all drug conjugation reactions. They catalyze glucuronidation, i.e., transfer of the glucuronic acid moiety from UDPGA onto nucleophilic hydroxyl, amine, carboxylic acid, thiol or thioacid groups of the substrates. There is a higher concentration of UGTs in the small intestine than in the liver, and expression levels of individual UGTs differ in these tissues [61].

**Figure 5:**
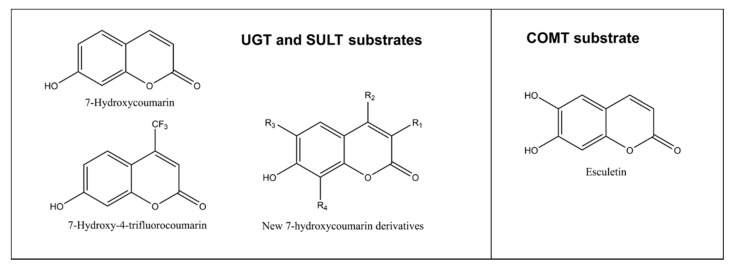
Structures of substrates conjugated to nonfluorescent metabolites. An exception is esculetin, which is weakly fluorescent but its 6-methoxy-7-hydroxycoumarin (scopoletin) is strongly fluorescent. New 7-hydroxycoumarin derivatives can have different combinations of substituents at position 3 (methyl, ethyl, 4-methylphenyl, 4-methoxyphenyl, 4-fluorophenyl, 4-hydroxphenyl, 4-dimethylaminephenyl, pyridine-3-yl or triazole), at position 4 such as methyl or trifluoromethyl, at position 6 such as methoxy or at position 8 such as methyl.

As discussed above, glucuronidation of the C7 hydroxyl group abolishes coumarin fluorescence (Figure 1B). Substituents at positions such as C3 or C4 do not quench the fluorescence of 7-hydroxycoumarin derivatives, but rather modify its intensity, depending on the substituent’s chemical nature (Figure 5). Based on this phenomenon, we developed a convenient quantitative multiwell plate assay to measure the glucuronidation rate of 7-hydroxycoumarin derivatives by human recombinant UGT enzymes. At 20 µM substrate concentration, the most active HFC glucuronidation catalysts were UGT1A10 followed by UGT1A6 >UGT1A7 >UGT2A1, whereas at 300 µM UGT1A6 was ~10 times better a catalyst than the other UGTs [62]. The activities of UGTs 1A3, 1A8, 1A9, 2B4 and 2B7 were low, whereas UGT1A1 and UGT2B17 exhibited no HFC glucuronidation activity. 

In a follow-up study [33] we constructed predictive homology models for the human UGT1A enzymes to design selective substrates for them. Six novel C3 substituted coumarin derivatives (4-fluorophenyl, 4-hydroxyphenyl, 4-methoxyphenyl, 4-dimethylaminophenyl, 4-methylphenyl or triazole) were synthesized. All tested compounds were glucuronidated to nonfluorescent conjugates by the intestinal UGT1A10. 4-dimethylaminophenyl and triazole C3 substituted 7-hydroxycoumarins were selective substrates for UGT1A10. The hydroxyl group of 7-hydroxycoumarin was oriented toward the cofactor UDPGA in the active site of the UGT1A10 model, indicating that there is space in the active site for an additional 6- or 5-ring substituent at position C3. The extensive fluorescence of these new 7-hydroxcoumarin derivatives as UGT1A10 substrates makes them suitable for quick and convenient activity measurements in a high-throughput format.

In a recent study [63] we used the multiwell plate assay to evaluate the glucuronidation characteristics of several 7-hydroxycoumarin derivatives in intestine and liver microsomes of beagle dog and human. High glucuronidation rates were observed in the liver and small intestine. The results underscored the high capacity of dog intestine for glucuronidation and revealed that different UGT enzymes mediate glucuronidation with distinct substrates selectivity in dog and human, even if all the substrates are 7-hydroxycoumarin derivatives.

#### 2.2.2. SULTs

Sulfonation by SULT enzymes takes place for nucleophilic phenols, alcohols or primary or secondary amines. The sulfate conjugates are negatively charged and more water-soluble than the parent compounds. These metabolites are readily excreted primarily to urine or sometimes transported to bile. Sulfonation and glucuronidation reactions act in a complementary way. Sulfonation as part of overall xenobiotic metabolism usually protects the body against adverse health effects. However, sulfate conjugates of hydroxylated amines such as heterocyclic amines can form reactive metabolites with detrimental consequences [61,64].

In analogy with glucuronidation, fluorescence is diminished by the sulfonation of 7-hydroxycoumarins (Figure 1B and Figure 5). We developed recently a fluorescence-based kinetic sulfonation assay using this principle, using the same 7-hydroxycoumarin substrates as in the glucuronidation assay described above [65]. Because this assay was sensitive and quantitative, it could be applied to determine sulfonation kinetics in liver cytosol of human, mouse, rat, pig, rabbit, dog and sheep. The assay can also be used to study potential inhibitors of sulfonation to reveal potential drug interactions.

#### 2.2.3. Catechol O-methyltransferases (COMTs)

COMT is the selective methylating enzyme of catechol structures, compounds having two hydroxyl substituents in adjacent positions of an aromatic ring. In humans, there is one COMT gene, from which a long membrane-bound and a short cytosolic COMT is expressed. Its activity can be measured with esculetin, a catecholic coumarin derivative [66,67]. The weakly fluorescent esculetin is methylated by COMT to either 6-methoxy-7-hydroxycoumarin (scopoletin) or 6-hydroxy-7-methoxycoumarin (isoscopoletin) (Figure 5). Isoscopoletin is nonfluorescent, and scopoletin is strongly fluorescent. Therefore, increase of fluorescence takes place in esculetin 6-O-methylation reaction, to which a convenient fluorescent-based assay for COMT activity is based. 

## 3. Applications

The two main lines of application for in vitro fluorescence-based assays are (1) measuring activities of XMEs in whole tissue samples or cellular fractions prepared from them or with recombinant or purified enzymes, and (2) using the assay as a test compound independent method to screen for potential inhibition of XMEs by new drug candidates.

The first application is the traditional one, used since the 1950s to measure activities especially of CYP enzymes, first in animals and later also in human tissues, since the 1960s in purified enzymes, and since the 1980s in recombinant enzymes. Quite probably, all tissues in mammals have been evaluated for CYP-catalyzed oxidation using fluorescence substrates, and research has extended to other organisms too. This research has generated a huge amount of data on oxidative metabolism in various organs and allowed for interspecies comparisons. The obvious shortcoming is the fact that only a few present fluorescence probes are truly CYP form selective, and virtually none are totally specific. We aim to overcome this shortcoming by using molecular modeling of XMEs, the flexible chemistry of coumarin, and testing new substrates with high-throughput in vitro assays. Examples of novel substrates are the UGT1A10 selective probes 3-(4-dimethylaminophenyl)coumarin and 3-triazolecoumarin [33]. We expect this approach to reveal ever more subtle features of interactions between coumarins and active sites of XMEs. 

The second application is more recent, arising from the need to detect CYP inhibition liability of drug candidates early in the drug discovery process. Numerous harmful interactions occur between drugs and other substances, including compounds in herbal products and food. Many such interactions are based on alterations of the plasma concentrations of a victim drug due to another compound causing inhibition and/or induction of the metabolic disposition of the victim drug. In the worst case, such interactions cause more than 10-fold increases or decreases in victim drug exposure, with potentially life-threatening consequences [32,68,69,70].

A major mechanism for clinically relevant pharmacokinetic interactions is inhibition of CYP enzymes. Various in vitro methods have been developed to identify and quantitate the type and extent of CYP inhibition. Fluorescence-based assays in high throughput are now routinely carried out to screen the inhibitory potencies of drug candidates and herbal constituents. Commercial kits containing recombinant CYPs, fluorescence substrates and other components needed for the reaction are available for such screens. The fluorescence-based approach for screening has several advantages: it is faster and much less expensive than LC-MS methods. Data can be generated quickly without compromising data quality [10,36,71,72].

CYP inhibition data form fluorescence assays are fairly consistent with findings from LC-MS methods, although variations for specific chemotypes are reported [27,73]. The rapid fluorescence methods still mostly have insufficient CYP form selectivity, and therefore incubations with individual recombinant CYPs are needed. Another limitation is the ability to identify multiple probe substrates suited to assess the inhibitory mechanism in case the test compounds have multiple binding regions at the active sites of several CYPs. In contrast with other pharmacokinetic assays used in drug discovery which monitor the fate of the test compound, CYP inhibition screens measure effects on the marker reaction, and therefore are test compound independent [36]. The novel coumarin substrates presented in this review would be especially suited for simple high-throughput testing of CYP inhibition properties of nonfluorescent compounds.

## 4. Conclusions

Coumarin and its derivatives have proven to be very useful ligands to many different types of XMEs, both as substrates and inhibitors. To evaluate catalytic activities of especially CYP enzymes and COMT, assays based on profluorescent coumarin substrates are sensitive, fast, reliable, simple, and low cost. The assay conditions can be easily optimized. The reverse phenomenon, measuring loss of fluorescence, can be applied in similar assay setups to measure activities of conjugating enzymes such as glucuronidation and sulfonation. These methods are especially well suited for laboratories with limited ability to invest in expensive analytical instrumentation. 

We and others have shown that it is possible to design coumarin derivatives with improved selectivity by molecular modeling approaches. This is important because thus far fluorescence substrates fail to accurately distinguish between different forms of CYPs and conjugating enzymes when measuring their activities in tissue samples. 

In drug development, fluorescence-based in vitro assays are standard methods used in inhibition screens since they give initial clues about the unwanted ability of test molecules to inhibit XMEs. These assays are robust and highly reproducible. The lack of enzyme form selectivity of substrates is no problem when recombinant enzymes are used. The same principle can be applied by investigators with less resources to obtain information about the ability of their compounds to interact with XMEs.

## Abbreviations

CYP	Cytochrome P450
XME	Xenobiotic-metabolizing enzyme
UGT	Uridine diphosphate glucuronosyltransferase
SULT	Sulfotransferase
LC-MS	Liquid chromatography-mass spectrometry
CEC	3-cyano-7-ethoxycoumarin
EFC	7-ethoxy-4-trifluoromethylcoumarin
MFC	7-methoxy-4-trifluoromethylcoumarin
MAMC	7-methoxy-4-aminomethylcoumarin
AMMC	3-[2-(N,N-diethyl-N-methylammonium)ethyl]-7-methoxy-4-methylcoumarin
BFC	7-benzyloxy-4-trifluoromethylcoumarin
UDPGA	Uridine 5’-diphosphoglucuronic acid
PAPS	3’-phosphoadenylyl sulfate
SAM	S-adenosylmethionine
BP	4-benzylpyridine
CBP	4-(4-chlorobenzyl)pyridine
HFC	7-hydroxy-4-trifluoromethylcoumarin
COMT	Catechol O-methyltransferase
